# Personality characteristics in emerging adults engaging in sex work: a systematic review

**DOI:** 10.1186/s40359-025-03242-6

**Published:** 2025-08-07

**Authors:** Leonie Carmen Bandurski, Christina M. Juchem, Saskia Baumgardt, Antonia Bendau, Eva Asselmann

**Affiliations:** 1 Department of Psychology, Institute for Mental Health and Behavioral Medicine, HMU Health and Medical University, Olympischer Weg 1, 14471 Potsdam, Germany; 2 Department of Psychiatry and Neuroscience, Charité – Universitätsmedizin Berlin, corporate member of Freie Universität Berlin and Humboldt Universität zu Berlin, CCM, Charitéplatz 1, 10117 Berlin, Germany

**Keywords:** Transactional sex, Escort, Prostitution, Webcam-sex, Pornography, Development

## Abstract

Personality may influence whether emerging adults do or do not engage in sex work. In turn, sex work may affect personality development during this sensitive developmental time frame. This systematic review synthesizes literature on personality and sex work in emerging adults. Seven of the ten identified studies investigated personality differences between individuals engaging vs. not engaging in sex work, four examined personality characteristics of sex workers only. Results suggest lower agreeableness, self-efficacy, and self-esteem and higher impulsivity, trait aggression, and antisocial traits among sex workers. The scant literature highlights the need for additional research to disentangle selection and socialization effects and examine within-person personality changes due to different types of sex work to inform targeted supportive interventions. This systematic review was pre-registered in the PROSPERO database for systematic reviews (2023, October 10, *registration number CRD42023402800*).

## Introduction

Sex work is associated with specific physical and psychological challenges. People involved in sex work interact sexually with people they do not know, which can increase the risk of sexually transmitted diseases, sexual violence, traumatic experiences, discrimination, and marginalization, especially in precarious employment [1]. In psychological research, selection effects refer to the idea that individuals with certain traits are more likely to enter specific environments or roles, whereas socialization effects describe how experiences within these environments can shape or change personality over time [2]. Applied to the context of sex work, personality may influence whether individuals engage in sex work (selection) and also change due to this type of work (socialization) [2]. In terms of selection effects, the likelihood of engaging in sex work may depend on certain personality traits and may be higher, for example, for individuals who are more risk-taking. In terms of socialization effects, personality may be affected by sex work experiences [2]. That is, sex work may have lasting effects on people’s thoughts, feelings, and behaviors over time, leading, for example, to greater risk-taking.

Entry into sex work often occurs early [3–5], not long after adolescence - a developmentally sensitive period that often relates to initial sexual experiences. Emerging adulthood, typically spanning ages 18 to 29, is marked by identity exploration, instability, and transitions in work, relationships [6]. These dynamics may contribute to increased vulnerability and risk-taking, which can intersect with entry into sex work – for example, in order to cope with occupational and financial uncertainty [7]. Research on the relationship between personality and sex work is therefore particularly relevant to emerging adults. However, how personality relates to sex work in emerging adults has rarely been investigated, and studies on this topic are fragmented across disciplines. The current study aims to systematically review and synthesize previous research on this topic, which is important for several reasons: First, it helps identify inconsistencies and open research questions to be addressed in the future. Second, key findings may inform targets for preventive interventions and outreach programs focusing on education and social support to reduce health risks and stressors and improve mental health. To date, no comprehensive review has synthesized the literature on personality and sex work, highlighting the need for a systematic overview of existing findings.

### Definition of sex work

Sex work refers to the exchange of sexual services for compensation, which may include money, goods, or other forms of payment [8]. It includes various forms of work, such as prostitution, transactional sex, escort services, webcam sex, or pornography. Sex work can take place online through video camera platforms or offline in the traditional form of face-to-face sexual intercourse (e.g., prostitution).

The role of personality may vary for different types of sex work. For example, traditional sex work (e.g., prostitution) relates to a higher risk of physical violence or contracting sexually transmitted diseases than online sex work (e.g., webcam sex), which relates to digital risks such as cybercrime or loss of online privacy [9]. Because of these contextual factors, different types of sex work need to be distinguished.

### The role of personality

Personality refers to individual differences in patterns of thoughts, feelings, and behaviors that are relatively stable across different situations and over time. Much of this variation between individuals can be described by structural models such as the Big Five [10] or the HEXACO model [11]. The Big Five traits include extraversion, conscientiousness, emotional stability, agreeableness, and openness to experiences, while the HEXACO model includes an additional sixth dimension, Honesty-Humility, which is characterized by sincerity, fairness, and modesty [11]. Broader conceptualizations of personality—such as McAdams’ integrative framework—go beyond trait models and also include characteristic adaptations (e.g., self-efficacy, locus of control, self-esteem, well-being, values, goals, or motivation). In this sense, personality is not limited to stable traits, but encompasses a wider range of psychological characteristics relevant to individual functioning [12].

Research has shown that personality can and does change over time. Evidence from the past two decades suggests that personality not only emerges during childhood and adolescence, but continues to develop throughout the lifespan, although non-volitional personality changes are typically small to moderate in adulthood [13]. A particularly sensitive developmental period is emerging adulthood, which on average is associated with increases in conscientiousness, agreeableness, and emotional stability [13, 14]. These developmental changes in personality traits have been linked to age-related major life events that involve changes in role demands, such as leaving home or entering the workforce [14, 15]. Similarly, entry into sex work can be conceptualized as a major life event related to specific role demands and potential stressors that may induce personality changes. For example, women in particular might become less agreeable after entering sex work to protect themselves from sexual assault or physical exploitation [16].

Personality may differ between individuals who engage in sex work and those who do not due to both selection and socialization effects: In support of selection effects, certain personality traits (e.g., high impulsivity, low self-esteem, low resilience) have been associated with an increased likelihood of engaging in sex work in emerging adulthood [17–19].

At the same time, personality may change as a result of sex work (socialization effects) [20]. Research shows that emotional intelligence and empathy often tend to increase in professions requiring emotional labor, such as clinical nurses [21] and psychotherapists [22]. These findings suggest that similar personality adaptations may occur in other emotionally demanding occupations, such as sex work, where attuning to clients’ emotional needs is a core aspect of the job [23].

Conversely, it is also plausible that sexual interactions with strangers or conflicts with clients, co-workers, and supervisors lead to decreases in these traits [24, 25]. For example, sex workers may become less agreeable to better protect and defend themselves when confronted with violence, abuse, discrimination, or marginalization. Such experiences and other stressors/threats (e.g., fear of sexually transmitted diseases) may also lead to lower emotional stability, self-esteem, self-efficacy, and/or interpersonal trust.

### Summary and objectives

This systematic review aims to synthesize previous studies on the following research questions: (1) Are there personality differences between emerging adults who engage in sex work and those who do not (between-person comparisons)? (2) Do personality traits differ between emerging adults who engage in different types of sex work (between-person comparisons)? (3) Is sex work during emerging adulthood associated with specific personality changes over time (within-person effects)?

## Methods

### Systematic literature search

This systematic review was conducted based on the PRISMA guidelines [26] and pre-registered in the PROSPERO database for systematic reviews (*registration number CRD42023402800*).

The systematic literature search took place from September 2022 to March 2023 using the databases EBSCOhost, Web of Science, and PubMed. The bibliographies of the identified papers were manually screened for additional papers. Peer-reviewed original research published in academic journals in English or German language was included. No restrictions were placed on publication date or other formalities. Search terms were applied to the title, keywords, and abstract of potential studies.

**Fig. 1:**
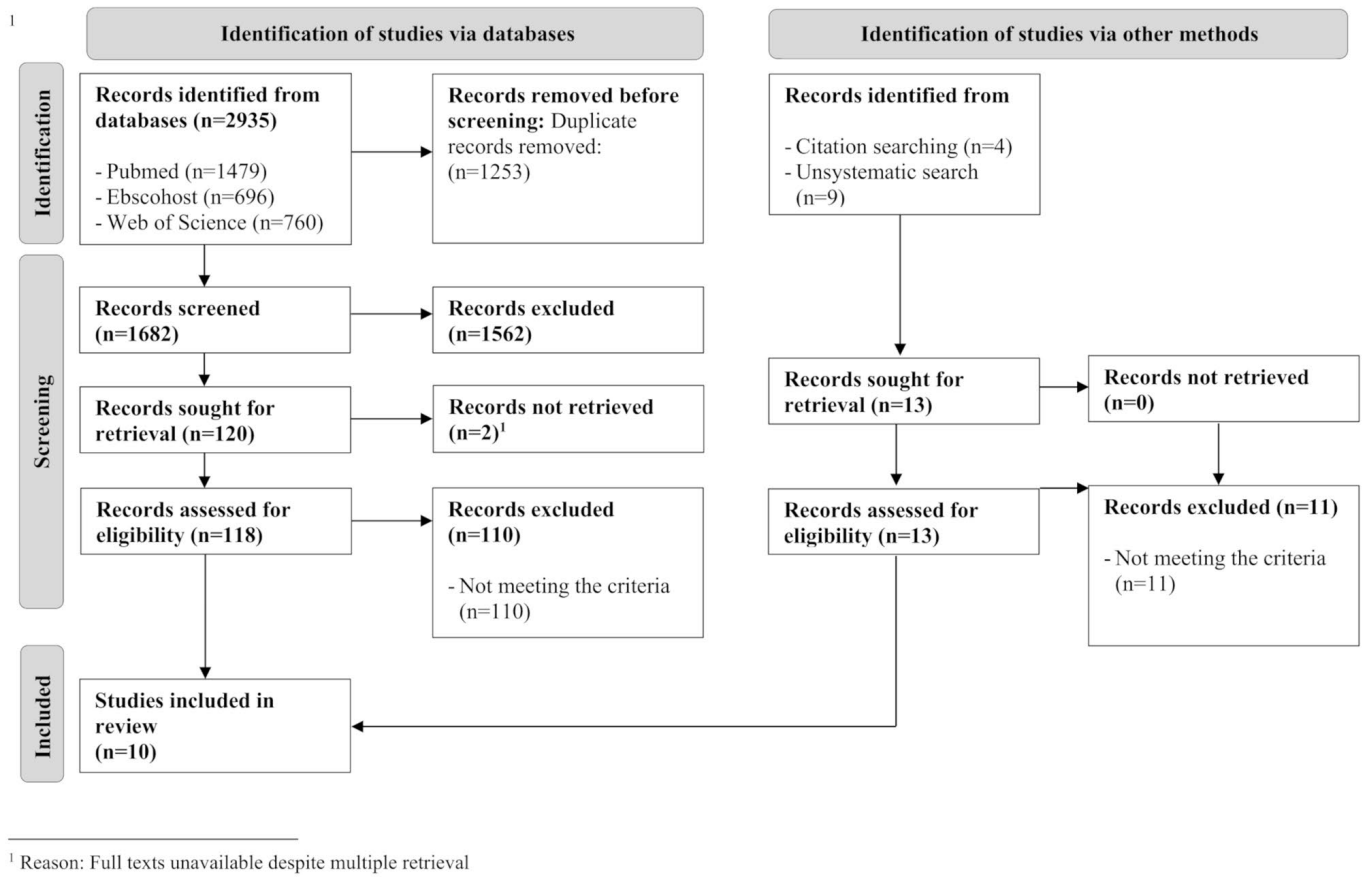
PRISMA flow diagram

The search terms (Table 1) included personality-related keywords (e.g., Big Five, locus of control, dark triad). In addition, search terms included sex work-related keywords (including all subtypes of sex work such as prostitution, escort, transactional sex, webcam performers, etc.) along with emerging adulthood keywords (e.g., university students, vocational students, adolescents, or young adults). The PRISMA flow diagram (Fig. 1) illustrates the screening, and data extraction procedures.

**Table 1:** Systematic review search terms

Subject Area	Search Terms
[adolescent-related terms]	( “Vocational training*” OR “vocational training student*” OR “vocational student*” OR apprentice* OR Apprenticeship OR student* OR undergraduate* OR college* OR universit* OR “university student*” OR campus OR young OR adult* OR “young adult*” OR adolescent* OR youth OR “emerging adulthood” OR “young adulthood” OR “Young people” OR adolescence OR “High school” OR graduates OR “High school graduate*” OR “high school student*” OR “secondary school” OR “secondary school student*” OR “senior high school” OR “senior high school student*” OR “high school senior*” OR “second chance education” OR “second course of education” OR “Secondary education” OR “second education” OR trainee OR “Professional training” )
	AND
[sexwork-related traits]	( “sex work” OR sexwork OR “sex worker*” OR “porn actor*” OR “porn-actor*” OR prostitut* OR “web-cam actor*” OR “web-cam-actor*” OR “selling sex” OR “paid sex” OR sexindustry OR “sex-industry” OR “sex industry” OR “transactional sex” OR “sugar daddy” OR “sugar baby” OR “call girl*” OR “call boy*” OR callgirl* OR callboy* OR escort* OR “street prostitut*” )
	AND
[personality-related traits]	( Personality OR “personality development” OR “personality change*” OR “personality trait*” OR “character strength*” OR “character trait*” OR “trait change*” OR Hexaco OR “Big Five” OR “five-factor model” OR openness OR agreeable* OR extraver* OR introver* OR neurotic* OR “emotional instability” OR “emotional stability” OR “emotionally stable” OR conscientious* OR “self-efficacy” OR mastery OR “well-being” OR “locus of control” OR “perceived control” OR “control belief*” OR “external control” OR “internal control” OR “self-esteem” OR motivation OR “dark triad” OR narcissis* OR Machiavelli* OR psychopath* OR “risk avers*” OR “risk affin*” OR “harm avoidan*” OR “novelty seek*” OR “Sensation seek*” OR “reward depend*” OR predictor* OR “risk factor*” OR “protective factor” OR “mental health” OR vulnerab* OR resilien* OR “behavioral inhibition” OR hardiness OR “mental toughness” OR temperament* )

### Screening and data extraction

The inclusion criteria were prespecified. Study selection screening was performed by two reviewers based on the inclusion criteria. One reviewer selected studies based on the inclusion/exclusion criteria, and a second reviewer reviewed this decision. In case of disagreement, a third independent reviewer was consulted, and a consensus decision was reached. The same procedure was followed for manually retrieved articles from the cited literature. The reviewers followed a standardized protocol for screening that involved several steps, including reviewing article titles and abstracts and working through the full texts of potentially relevant articles.

From the studies that met the inclusion criteria, information on study methodology and characteristics, including country, type of sex work, personality parameters, sample size, and study design, was collected. Studies were included based on several criteria: First, although “emerging adulthood” is typically defined as ages 18–29 [6], we did not apply a strict age range due to variability across studies, some of which included broader age ranges still consistent with emerging adulthood characteristics. Second, we considered any form of sex work, current or past, was considered, including street-based, escorting, and online sex work. Third, only studies using validated personality measures (e.g., Big Five Inventory) were included. Finally, studies were required to needed to report statistical measures such as correlations, effect sizes, or difference scores.”

In addition, sociodemographic characteristics of study participants were recorded, as were the main objectives, main outcomes, including information on statistical significance and effect sizes. Aspects of reliability and validity, limitations, and possible biases were extracted and aggregated. We also applied the Newcastle-Ottawa Quality Assessment Scale [27], adapted for cross-sectional studies [28], and report the quartile rankings of the journals in which the included studies were published to assess their quality.

## Results

In total, 10 studies met the inclusion criteria. Out of these, 9 studies were based on cross-sectional data, 1 study used a longitudinal design with 3 waves [29]. All studies, including their methods, results, and effect sizes, are presented in Table 2.

**Table 2:** Included studies (total *N* = 10 studies)

Author	Year	Country	Sample	Study Design	Sex work	Personality Construct and Measure	Key Finding	Research Questions Addressed
Averdijk et al.	2019	Switzerland	*N* = 1,675 adolescents (including *N* = 1197 involved in sex work) (48% female, 52% male; age at wave 1 *M* = 13.7 years, *SD* = 0.37, at wave 2 *M* = 15.4 years, *SD* = 0.36, at wave 3 M = 17.4 years, *SD* = 0.37, age range not specified)	Longitudinal, 3 waves over a timespan of ∼ 4 years	Trading sexual services over the last 2 years (i.e., oral, anal, or vaginal sex, touch intimate body parts, photograph, or film naked)	Optimism (developed by the z-proso team, *no reference listed*), Self-efficacy (adapted from [30]), Self-control (adapted from [30], Generalized trust (adapted from the World Value Survey, [ 31]), Shame and guilt (adapted from [32]).	Logistic regressions were conducted to test whether variables at ages 13–15 predicted selling sexual services at ages 15–17.^a^ Lower self-efficacy predicted a higher likelihood of selling sexual services (*OR* = 0.33). Optimism, self-control, shame, and guilt were not significantly associated with the likelihood of selling sexual services (*p*-values > 0.05).	RQ1
Betzler et al.	2014	Germany	*N* = 4,386 university students (including *N* = 227 involved in sex work) (44% female, 42% male, 14% did not specify gender, age *M* = 24.4 years, *SD* = 3.7, age range not specified) ^a^	Cross-sectional	Past or present involvement in sex work	Neuroticism, extraversion, openness, agreeableness, conscientiousness (Big Five Inventory by [33]	T-test were used to compare students who had vs. had not been involved in sex work. Agreeableness was lower in students who had vs. had not been involved in sex work. Neuroticism, extraversion, openness, and conscientiousness did not differ significantly between both groups (*p*-values > 0.001).	RQ1
Blum et al.	2018	United States	*N* = 1,453 university students (including *N* = 30 involved in sex work) (26% male, 63% female, 11% other, *age not specified*)	Cross-sectional	History of selling sex (i.e., oral sex, penetrative sex, manual stimulation, performance of sex acts for another person)	Self-esteem (Rosenberg Self-Esteem Scale (RSES), [34] Impulsiveness (i.e., 3 facets: attentional, motor, and nonplanning; Barratt Impulsiveness Scale, Version 11, [35]. Compulsivity (Cambridge–Chicago Compulsivity Trait Scale; [36]	T-test were used to compare individuals who had vs. had not sold sex.^a^ Low self-esteem (i.e., score < 15) was more prevalent in students who had vs. had not sold sex (*V* = 0.10). Attentional impulsiveness was higher in students who had vs. had not sold sex (*g* = 0.53) Motor/non-planning impulsiveness and compulsivity did not differ significantly between both groups (*p*-values > 0.01).	RQ1
Chen et al.	2017	China	*N* = 457 female sex workers (age *M* = 25.15 years, *SD* = 5.91, ranging from 18 to 42 years)	Cross-Sectional	Sample consisted of sex workers recruited from commercial sex venues (e.g., bars, karaoke, massage parlors)	Negative trust (Propensity to Trust Survey; [37]; Thwarted belongingness (Interpersonal Needs Questionnaire; [38]	Linear regressions and mediation analyses were conducted to test the association between negative trust, thwarted belongingness, and depressive symptoms. Higher negative trust predicted higher depressive symptoms (*β* = 0.30). Higher thwarted belongingness predicted higher depressive symptoms (*β* = 0.50). Higher negative trust predicted higher thwarted belongingness (*β* = 0.27). Higher thwarted belongingness partially mediated the relationship between negative trust and depressive symptoms (*R*^*2*^ = 0.07; F(1, 434) = 34.65, *p* <.001).	None (within-group only)
Edwards et al.	2022	United States	*N* = 1,250 university students involved in sex work (64% female, 36% male, age *M* = 19.96 years, *SD* = 1.55, age range not specified)	Cross-sectional	Engagement in erotic services (Erotic Activity Questionnaire, EAQ)	Psychopathic traits i.e., interpersonal-affective and impulsive-antisocial (Self-Report Psychopathy Scale-III; [39]	Logistic regressions were conducted to test whether psychopathic traits predicted provision of erotic services. Higher impulsive-antisocial traits predicted provision of erotic services (*OR* = 5.82). Interpersonal-affective traits were not significantly associated with provision of sexual services (*p*-values > 0.01).	RQ1
Ernst et al.	2021	Germany	*N* = 4,386 university students (32% male, 44% female, 14% did not specify gender, age *M* = 24.4 years, *SD* = 3.7, age range not specified) ^a^	Cross-sectional	Offering sex work (i.e., prostitution, escort services with or without sexual intercourse, striptease, and webcam, phone sex or self-specified)	Happiness (from the German Socio-Economic Panel (SOEP), [40]	Mann–Whitney U test and cross-tabulations were used to compare students offering sex services to age-matched students not offering sex services. Both groups did not differ significantly in happiness during the past 3 months (*p*-values > 0.05).	RQ1
Kinzl et al.	2010	Germany and Austria	*N* = 99 female university students engaging in sex work (age *M* = 23 years, ranging from 19–32 years)	Cross-sectional	Sample consisted of sex workers recruited online	Personality styles (Persönlichkeits-Stil-und Störungs-Inventar; [41]	Rates of elevated scores (T-values > 60) on different personality styles were reported: 60% for ‘self-determined – antisocial’, 47% for ‘amiable – histrionic’, 43% for ‘ambitious – narcissistic’, 40% for ‘self-willed – paranoid’, 36% for ‘critical – negativistic’, 32% for ‘optimistic – rhapsodic’, 27% for ‘reserved – schizoid’, 27% for ‘meticulous – compulsive’.	None (within-group only)
Lung et al.	2004	China	*N* = 158 female adolescents engaging in sex work (age *M* = 15.6 years, *SD* = 1.5; age range not specified) *Control group*: *N* = 65 female adolescents (age *M* = 15.51, *SD* = 0.66)	Cross-sectional	Sample consisted of sex workers living in a halfway house for adolescent prostitutes	Neuroticism, extraversion, psychoticism (Junior Eysenck Personality Questionnaire, adapted from [42]	T-test were used to compare adolescent sex workers to an age-matched control group. There were no significant differences in neuroticism, extraversion, and psychoticism between both groups (*p*-values > 0.01).	RQ1
Mo et al.	2018	China	*N* = 87 female sex workers (age *M* = 20.89 years, *SD* = 2.54, ranging from 16–25 years)	Cross-sectional	Sample consisted of sex workers recruited by an NGO for prevention of sexually transmitted diseases	Self-efficacy (adapted from Hoeppner, 2011), Hope (Snyder State Hope Scale; [43]	Linear regressions were conducted to test associations of self-efficacy and hope with mental health. Higher self-efficacy (*β* = 0.27) and higher hope (*β* = 0.20) predicted better mental health in female sex workers.	None (within-group only)
Nasir et al.	2012	Malaysia	*N* = 42 female adolescent sex workers (age *M* = 18.19 years, *SD* = 1.63, ranging from 15–21 years)	Cross-sectional	Sample consisted of sex workers recruited from various location (e.g., prison, massage parlors, streets)	Self-esteem	Pearson correlation analyses yielded a negative correlation between cognitive distortion and self-esteem (*r* =.53), and a negative correlation between depression and self-esteem (*r* =.52).	None (within-group only)

^a^ Statistical significance was defined as *p* <.01

### Samples

Sample sizes in the included studies varied between 42 [44] and 4,386 [45] individuals. In line with the specified search criteria, the samples consisted of adolescents and emerging adults. Participants were recruited from China, Malaysia, the United States, Germany, Switzerland, and Austria. Participants’ age ranged from 13.7 years (SD = 0.37) [29] to 25 years (SD = 5.91) [46]. Participants’ age ranged from 13.7 years (SD = 0.37) [28] to 25 years (SD = 5.91) [45]. One study [16] reported a wider age range (18–42 years, M = 25.15, SD = 5.39); however, this sample was retained because the mean age was still close to the upper bound of emerging adulthood, and because the study focused on a transitional life phase (college/university) that is often associated with emerging adulthood regardless of chronological age.

In three studies, participants were actively recruited from sex work facilities. Six studies drew their samples from university or schools, and one study drew a convenience sample from various locations including prisons, holding cells, and rehabilitation centers (Table 2).

### Measures of sex work

In all ten studies, sex work was measured via self-report. Participants indicated whether they were currently engaged in sex work or had engaged in sex work in the past. Type of sex work varied between studies (e.g., oral sex, penetrative sex, prostitution, webcam sex, escort services) and was not always specified.

### Personality

The included studies focused on the Big Five and other personality traits (e.g., self-efficacy, self-esteem, impulsivity, aggression, psychoticism, and optimism), assessed with self-report questionnaires such as the Big Five Inventory [33] or the Rosenberg Self-Esteem Scale [34].

***Big Five***. Two studies examined differences in the Big Five between individuals who did vs. did not engage in sex work [45, 47]. In these studies, no group differences were found, except that university students who were/had engaged in sex work were less agreeable compared to those without sex work experiences [45].

***Other traits***. Nine studies investigated associations between sex work and other personality traits in emerging adults. In adolescents, lower self-efficacy (but not self-control, optimism, shame, and guilt) prospectively predicted a higher likelihood of selling sexual services four years later [29]. In university students, self-esteem was lower and attentional (but not motor and non-planning) impulsiveness was higher in those with vs. without a history of selling sex [17]. Happiness in the past three months did not differ significantly between university students who did vs. did not provide sex work [48]. Psychoticism did not differ between adolescent sex workers and age-matched controls [47]. Regarding psychopathic traits, impulsive-antisocial traits but not interpersonal-affective traits were higher in undergraduate students who did vs. did not provide erotic services [18].

Four studies investigated personality characteristics in sex workers only (i.e., without direct comparisons to individuals not engaging in sex work). One study on female university students in Germany and Austria reported elevated scores of the ‘antisocial’ personality style in 60% of individuals, the ‘histrionic’ style in 47%, the ‘narcissistic’ style in 43%, and the ‘paranoid’ personality style in 40% of individuals [49]. Higher levels of negative trust predicted increased depressive symptoms, partially mediated by higher levels of thwarted belongingness in sex workers in Western China [46]. Both self-efficacy and hope was linked to better mental health in young female sex workers in Hong Kong [50]. Finally, a negative correlation between cognitive distortion and self-esteem, as well as, between depression and self-esteem was reported in samples of Malaysian sex workers in emerging adulthood [44].

### Quality assessment

To assess the quality of the included studies, the Newcastle-Ottawa Quality Assessment Scale (adapted from [28]) was applied to all extracted studies. Two of the included studies were of “good quality” according to the Newcastle-Ottawa framework, and 8 studies were of “poor quality” according to the Newcastle-Ottawa framework with a very high risk of bias (according to the interpretation criteria of [27]) (Table 3). In terms of quartile rankings, 7 studies were published in Q1 journals, while 1 study was published in a Q2 journal, 1 study in a Q3 journal, and 1 study in a Q4 journal.

**Table 3:** Newcastle-Ottawa quality assessment Scale^1^ [28] and quartile rankings

First author	Year	Selection (Maximum 5 stars)				Comparability (Maximum 2 stars)	Outcome (Maximum 3 stars)		Total Quality Score^2^	Quartile rankings^3^
		Representativeness of the sample	Sample size	Non-Response rate	Ascertainment of the exposure	Confounding factors are controlled	Assessment of the outcome.	Statistical analyses		
Averdijk	2019	*	-	-	**	*	*	*	6 stars	Q1
Betzler	2014	*	-	-	**	-	*	*	5 stars	Q1
Blum	2018	*	-	-	**	-	*	*	5 stars	Q1
Chen	2017	*	-	-	**	-	*	*	5 stars	Q1
Edwards	2022	*	-	-	**	-	*	*	5 stars	Q1
Ernst	2021	*	-	-	**	-	*	*	5 stars	Q2
Kinzl	2010	*	-	-	**	-	*	*	5 stars	Q3
Lung	2004	*	-	-	**	-	*	*	5 stars	Q1
Mo	2018	*	-	-	**	-	*	*	5 stars	Q4
Nasir	2012	*	-	-	**	-	*	*	5 stars	Q1

Note. ^1^ The Newcastle-Ottawa Scale (NOS), adapted for cross-sectional studies, rates methodological quality across three domains with a maximum of 10 stars (1) Selection (max. 5 stars): Evaluates the representativeness of the sample, sampling method (e.g., random or complete), response rate reporting, validity of exposure measurement (e.g., use of validated personality scales), and whether a sample size justification or power analysis was provided. (2) Comparability (max. 2 stars): Assesses control for confounding variables, awarding one star for controlling key confounders (e.g., age, socioeconomic status) and a second star for additional relevant covariates. (3) Outcome (max. 3 stars): Considers whether outcomes were measured using validated tools, whether all participants were assessed similarly, and whether appropriate statistical analyses (e.g., confidence intervals, significance tests) were conducted [27]

^2^ “good quality”: 3 or 4 stars in selection domain AND 1 or 2 stars in comparability domain AND 2 or 3 stars in outcome/exposure domain / “fair quality”: 2 stars in selection domain AND 1 or 2 stars in comparability domain AND 2 or 3 stars in outcome/exposure domain / “poor quality”: 0 or 1 star in selection domain OR 0 stars in comparability domain OR 0 or 1 stars in outcome/exposure domain (according to the interpretation criteria of [27]

^3^ Q1 refers to the top 25% of journals, Q2 refers to the 25–50% group, Q3 refers to the 50–75% group, and Q4 refers to the 75–100% group. The most prestigious journals within a subject area are those in the first quartile, Q1 [51]. We refer to quartile rankings form Clarivate in 2023

## Discussion

The aim of this systematic review was to synthesize previous studies on the association between personality and sex work in emerging adults. Overall, few studies on this topic were identified: Only 7 studies focused on between-person personality differences in emerging adults who did vs. did not engage in sex work. Four studies investigated personality characteristics of sex workers only. There was not research on personality differences between emerging adults engaging in different types of sex work or on within-person personality changes related to sex work. Thus, no conclusions can be drawn regarding research questions (2) and (3). Furthermore, the quality of the included studies was low in terms of sample selection, comparability of groups, and results. Most of the excluded studies either focused on adults engaged in sex work outside the emerging adulthood age range or investigated clinical parameters rather than personality traits, which were the primary focus of our review.

Regarding research question (1), the identified studies provided little evidence of Big Five differences between emerging adults who engaged in sex work and those who did not [50], with the exception of one finding that agreeableness was lower in those who sold sex [45]. These results for agreeableness are consistent with findings from older samples [52] and may reflect selection effects: individuals who care less about social norms may feel less inhibited about working in a stigmatized occupation. Sex workers may also tend to experience greater interpersonal stress (e.g., due to conflict with or antipathy toward clients), which may reduce their capacity to behave kindly and altruistically toward others. The fact that there was little evidence for other Big Five differences associated with sex work in emerging adults is inconsistent with some findings from older adults that levels of extraversion, conscientiousness, and/or openness were higher for individuals who sold sex than for those who did not [23]. A possible explanation for this inconsistency is that younger adults, in particular, may often engage in sex work due to contextual factors (e.g., financial constraints), so that Big Five traits may play a negligible role.

In terms of other personality traits, some studies found that emerging adults with lower self-efficacy were more likely to enter sex work [29] or that self-esteem was lower for emerging adults who sold sex than for those who did not [17]. These results are consistent with findings in (older) adults that self-esteem was lower [53, 54] between those who did vs. did not engage in sex work, however no significant differences have also been reported [55]. Some researchers even found that sex work had both positive and negative effects on self-esteem [56] or that self-esteem was higher in more vs. less experienced sex workers and concluded that sex work might strengthen self-esteem (e.g., by providing a sense of autonomy, control, and financial independence) [57]. Given these results, one could speculate whether sex work has initial destabilizing effects on mental health and related personality traits (e.g., self-esteem) – especially in young and less experienced adults.

The identified studies also found that antisocial traits [18, 49] were higher for emerging adults who engaged in sex work than for those who did not. These findings are consistent with evidence from older samples that levels of impulsivity [58] or impulsive sensation seeking [59] were higher among people who sold sex. A possible explanation for this result is that higher levels of impulsivity and sensation seeking are related to higher levels of risk taking in various domains, including career decisions [60].

Overall, the findings on personality differences may be due to both selection and socialization effects. On the one hand, individuals with certain personality traits may be more likely to enter sex work (selection effect). For example, individuals who are less agreeable may be more likely to become sex workers despite potential victimization, discrimination, or marginalization. On the other hand, certain behavioral expectations and experiences associated with sex work may lead to personality changes over time (socialization effect). For example, sex workers may become less agreeable if they are repeatedly exposed to challenging interpersonal situations (e.g., sexual abuse or physical exploitation). Further longitudinal studies with repeated personality assessments before, during, and after sex work are needed to test these assumptions and to disentangle selection and socialization effects.

### Quality of the included studies

We assessed the methodological quality of the included studies using the Newcastle-Ottawa Scale, a widely acknowledged tool for evaluating observational research. One author performed a quality assessment using the Newcastle-Ottawa Scale, and ambiguous cases were reviewed with the senior author to ensure consistency. Based on this framework, eight of the ten studies were rated as having low methodological quality. Common limitations involved sample selection (e.g., lack of power analyses, unreported response rates), group comparability (e.g., no control for potential confounders or common biases), and outcome assessment (e.g., exclusive reliance on self-report measures).

For instance, most studies did not account for potential confounding variables such as socioeconomic status, cultural background, or early traumatic experiences—factors that may, in part, explain the observed negative associations between sex work and self-esteem [61–63].

It is important to note, however, that the Newcastle-Ottawa Scale was originally developed for cohort and case-control designs. Its application to cross-sectional or non-randomized studies, as in the present review, may therefore be limited and should be interpreted with appropriate caution.

Notably, 62.5% of the studies rated as low quality were published in Q1 journals, suggesting that editors and peer reviewers nonetheless recognized their scientific relevance and the contextual challenges associated with research in this field.

Indeed, empirical studies on sex work face unique methodological barriers, including stigma, restricted access to participants, small or hidden populations, high dropout rates, and selection bias. These structural constraints likely contributed to the lower methodological ratings and underscore the need for more rigorous study designs.

Given the limited number of available studies and their heterogeneity in terms of measures, contexts, and age groups, our findings should be interpreted with considerable caution. They do not allow for firm generalizations about personality traits among emerging adults engaged in sex work.

Moving forward, the field would greatly benefit from high-quality, longitudinal research using standardized and validated instruments. Methodological challenges could be addressed, at least in part, by implementing snowball sampling and partnering with community-based organizations, which may help to build trust and improve both participant recruitment and retention.

### Strengths and limitations

This review has several strengths: First, it addresses an understudied and socially relevant topic by systematically synthesizing research on the relationship between personality traits and sex work in emerging adults. Second, it applies a rigorous and transparent methodological approach, including a pre-registered protocol, comprehensive search strategy, and standardized quality assessment (adapted Newcastle-Ottawa Scale). Third, it provides theoretical and practical implications by highlighting personality-related factors that may be relevant for both selection into and socialization through sex work.

However, our study is not without limitations: We only included studies published in English or German and excluded studies published in other languages. Therefore, generalizability to non-English speaking research communities and populations may be limited. Furthermore, we were not able to exclusively focus on emerging adults because some samples were characterized by a broader age range, including middle-aged adults. All of the included studies were based on self-report, which may be subject to social desirability, memory, and reporting biases. While acknowledging these limitations, our systematic approach contributes a structured synthesis of prior findings and identifies important avenues for future research.

## Conclusions

This review highlights a striking gap in the literature: research on the relationship between personality and sex work among emerging adults remains scarce. The available evidence suggests that individuals who engage in sex work may experience lower levels of agreeableness, self-efficacy, and self-esteem, and higher levels of impulsivity, trait aggression, and antisocial tendencies than those who do not.

To better understand these patterns, future research is needed to disentangle selection effects (i.e., personality traits predicting sex work) from socialization effects (i.e., personality changes resulting from sex work). Longitudinal studies could also help clarify whether and how sex work is associated with within-person personality development over time.

Future studies should prioritize methodologically rigorous designs, including pre-registration, sample size planning, and multi-method approaches that go beyond self-reports—such as incorporating informant ratings or behavioral observations. Moreover, the type of sex work, as well as individual factors (e.g., gender) and contextual influences (e.g., legal frameworks), should be explicitly considered.

Notably, none of the included studies examined age or gender differences. Male and female sex workers may face gender-specific norms, risks, and stressors, potentially resulting in distinct personality trajectories. Similarly, younger and older sex workers may encounter different pressures—ranging from age-related stigma and physical demands to varying career expectations—which could influence both personality and mental health in age-specific ways.

Legal status also plays a critical role: For example, in the U.S., sex work is mostly illegal except in some counties, while webcam work and pornography are generally allowed. In Germany, Austria, and Switzerland, sex work is legal, but age of consent laws may restrict younger emerging adults from participating legally even where sex work is permitted. Criminalization may lead to added burden such as fear of prosecution, social isolation, or reduced access to health care, making country-specific effects plausible.

In addition, issues of consent are particularly salient for emerging adults, as this age group may be more vulnerable to coercion, exploitation, or limited autonomy due to socioeconomic pressures. Future research should therefore also examine how consensual versus non-consensual entry into sex work shapes personality-related outcomes. In such settings, behavioral observations could still be feasible if conducted within supportive, low-threshold environments such as counseling or outreach centers that work in close collaboration with sex workers and uphold strict ethical standards.

Assuming future research replicates the current findings, emerging adults who engage in sex work could benefit from targeted interventions aimed at strengthening self-efficacy, self-esteem, emotion regulation, and interpersonal competencies. Such interventions may support not only personality development but also mental and physical health and broader life course outcomes. For instance, promoting self-esteem in young sex workers could help reduce psychological distress and health risk behaviors. In line with this, studies have found that higher self-efficacy is associated with better mental health outcomes among adult sex workers [50]. Additionally, these interventions may offer practical strategies to disengage from involuntary sex work, protect against violence, or cope with stigma [56].

## Data Availability

No datasets were generated or analysed during the current study.
